# Relative validation of a pre-coded food diary in a group of Norwegian adults – Comparison of underreporters and acceptable reporters

**DOI:** 10.1371/journal.pone.0202907

**Published:** 2018-08-30

**Authors:** Jannicke Borch Myhre, Anne Marte Wetting Johansen, Anette Hjartåker, Lene Frost Andersen

**Affiliations:** Department of Nutrition, Institute of Basic Medical Sciences, University of Oslo, Oslo, Norway; University of Cape Town, SOUTH AFRICA

## Abstract

Estimating dietary intake is important for both epidemiological and clinical studies. In large studies, a balance has to be achieved between methods with high accuracy and methods that are easy to use. The aim of the present study was to compare results from a pre-coded scanable food diary (PFD) with results from a weighed record (WR) in a group of Norwegian adults. We also explored differences in day-to-day energy intake and the distribution of energy intake across the day in acceptable reporters (ARs) and underreporters (URs). Participants (n = 114, mean age 35 years, 68% women) recorded dietary intake with the PFD for 7 consecutive days. One week after completing the PFD, participants completed a 7 days WR. No difference in mean energy intake was seen between methods. Few differences were seen for the macronutrients, the most noticeable difference being the percentage of energy (E%) from carbohydrates which was significantly lower with the PFD (47 E%) than with the WR (49 E%). For the micronutrients, intakes of calcium and vitamin A were both significantly higher with the PFD than with the WR. Pearson’s correlation coefficient ranged from 0.47 (tocopherol) to 0.76 (E% carbohydrates) for all nutrients. Bread intake was significantly lower with the PFD while the intakes of edible fats, cheese and beverages were higher. Twenty-eight percent of the participants were found to be URs with the PFD. No clear pattern of underreporting at certain recording days or times of the day was seen. In conclusion, the results showed similar energy intakes and few differences in food and nutrient intakes between the PDF and the WR at the group level. Somewhat larger differences between the methods were seen at the individual level. Because of the reduced work load on both participants and researchers, the PFD seems a suitable alternative to the WR.

## Introduction

Estimating dietary intake is important for both epidemiological and clinical studies. When evaluating associations between dietary intakes and health, errors in diet-report instruments can result in important diet-disease relationships being either erroneously identified or overlooked [1–3]. In large studies, a balance has to be achieved between methods with high accuracy and methods that are easy to use. The food record method gives an open ended and detailed dietary assessment, but the major obstacle to using food records in large population surveys has been the huge, costly workload linked to coding (e.g. allocating database codes to recorded foods) and controlling coded data in addition to the considerable work burden on the participants. The food frequency questionnaire is far less time consuming for both researchers and participants, but has limited possibility to assess intake at the individual level [4]. An easy-to-use instrument is needed in order to obtain acceptable results with reduced burden both for the participants and for the researchers. A scanable pre-coded food diary (PFD) using household measurements and photographs for portion size estimation could substantially reduce costs and simplify the work for the participants by eliminating or reducing the need for measuring portion sizes and writing [5]. A PFD was developed for use in a Norwegian nationwide dietary survey among children and adolescents [6] and has later been revised for use among adults. The aim of the present study was to validate the intakes of energy, 17 nutrients and 14 main food groups estimated by the PFDs by using weighed records (WR) as the reference method in a group of Norwegian men and women. As different statistical approaches reflect different aspects of validity, results from multiple statistical techniques will be presented.

Misreporting of energy intake is a serious problem in dietary studies [7–9]. To learn more about the pattern of underreporting, we also compared day-to-day energy intake as well as distribution of energy intake across the day in participants classified as underreporters (URs) and acceptable reporters (ARs).

## Materials and methods

### Design

Data collection started in 2002 and ended in 2004. The participants recorded their diet for seven consecutive days using the PFDs. One PFD was filled in for each day of the recording period. While recording their diet using the PFD, most of the participants also wore the validated position-and-movement monitor ActiReg® [10]. The ActiReg® uses a combined second-to-second recording of body position and motion to calculate energy expenditure. Results from the ActiReg® registration will be published separately. One week after completing the seven days record with the PFD, participants started a seven days WR. The participants were instructed to maintain their normal eating habits throughout the recording periods. Feedback letters with data on individual energy and nutrient intake were sent to each of the participants after the study was completed.

### Subjects

**Fig 1:**
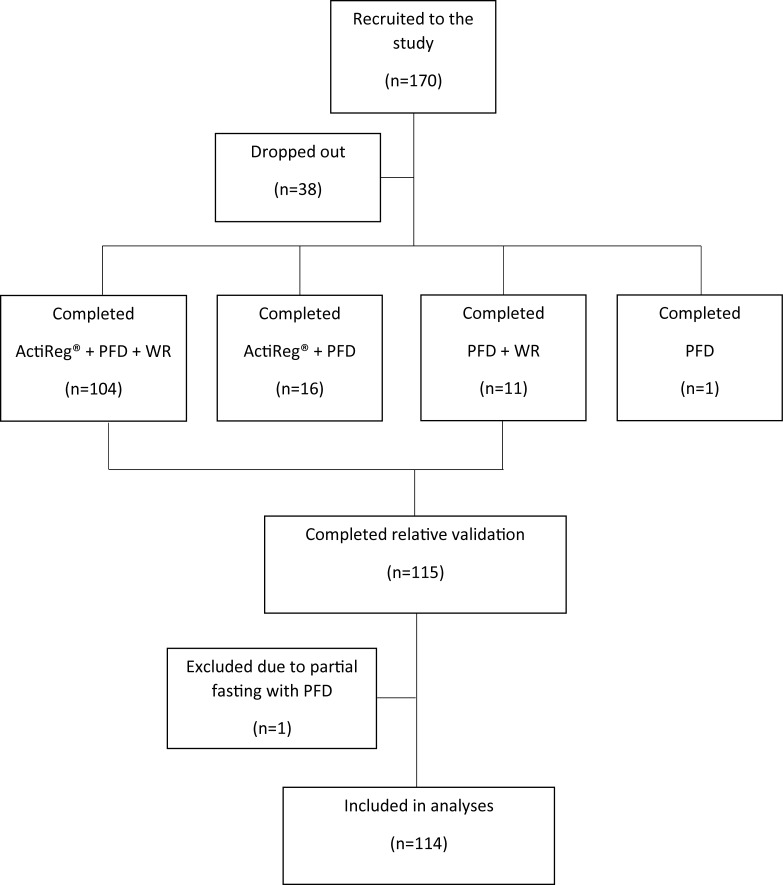
Flow chart of study participation. PFD, precoded food diary; WR, weighed record.

Subjects were recruited by posters or at personal request from fitness centers in the Oslo area, medical students at the University of Oslo, students from Oslo University College, employees and friends and family of employees of the Department of Nutrition, University of Oslo, employees from the Norwegian army and employees from the Norwegian Food Safety Authority. As the ActiReg® could not be used in water and was not able to record energy expenditure from weight bearing exercise, participants who took part in swimming or strength exercise more than 3 times per week could not be included in the study. No other exclusion criteria were set. A total of 170 men and women volunteered to participate in the study (Fig 1). Thirty-eight (22%) dropped out, and 132 completed the PFD. Of these, 115 also completed the WR. One woman was excluded due to partial fasting during the period of recording with the PFD. Hence, 114 participants (37 men and 77 women) were included in the analyses. The study was approved of by the Regional Committees for Medical and Health Research, Region South (reference number S-02132) and by the Norwegian Center for Research Data (reference number 200200623). Written informed consent was obtained from all participants.

### Pre-coded food diary (PFD)

The 18 page PFD used in this study comprises 294 pre-coded food alternatives grouped together according to a typical Norwegian meal pattern [11]. Each food group is supplemented with open-ended alternatives. The design of the booklet is similar to a cross-table with foods listed on the left and time span across the top. Each day is divided into five time spans, of which four time spans cover four hours each, e.g. from 06:00 to 10:00, from 10:00 to 14:00 etc., and one time span covers eight hours from 22:00 to 06:00. Food amounts are presented in predefined household units (e.g. spoons, dl, etc.) or as portions estimated from photographs in a photographic booklet. The photographic booklet contains color photographs of two to four portion sizes for 15 food items and has been validated in a group of children and adolescents aged 9 to 19 years with acceptable results [12]. The participants recorded food items by filling in how many units they had consumed in the correct time span. The PFDs were to be filled in either immediately after finishing a meal, or foods could be recorded on attached notepaper and entered into the diary in the evening. The PFD takes about 10–15 minutes per day to fill in [13]. Oral instructions were given in person or in small groups, and all participants received written information about the use of the diaries.

### Weighed record (WR)

The participants were provided with a digital scale (precision ±1 g, maximum capacity 2500 g) and a booklet for recording dietary intake for seven consecutive days. Written information and in person instructions were given, and the participants were instructed to weigh and record all foods and drinks that they consumed during the WR period. Brand names and descriptions of methods of food preparation and recipes for composite dishes were to be included whenever possible. For foods and drinks consumed outside the home, participants were asked to give as much detail as possible about the food items as well as estimates of portion sizes.

### Nutrient and food group analysis

The PFDs were scanned using Teleform version 6.0 software (Datascan, Oslo, Norway), and the open ended alternatives were manually coded. All recorded foods from the WR were manually coded by two trained nutritionists, and all entries were proofread by both nutritionists to avoid differences in coding practices. Daily intakes of energy, nutrients and food groups were calculated using a software system (KBS, version 3.1, 2002) developed at the Department of Nutrition, University of Oslo. The food database is based on the Norwegian Food Composition Table and is continuously supplemented with data on new food items. The main content of the presented food groups is described in Table 1. Dietary supplements were excluded from all calculations.

**Table 1:** Description of the presented food groups.

Food group	Description
Bread	white bread, semi-dark bread, dark bread, unspecified bread, crisp bread, tortillas, crackers
Cereals	flour, dry rice, dry pasta, sweetened breakfast cereals, unsweetened breakfast cereals, pizza
Cakes	yeast leavened bakery products, cream cakes, cookies, other cakes
Potatoes	fresh potatoes, potato powder, French fries
Vegetables	fresh or frozen vegetables, canned vegetables, dry legumes
Fruits and berries	fresh fruits and berries, dried fruit, canned fruit, jams and marmalades, juice, concentrated cordials
Meat	meat, whole pieces*, ground meat *, salted meat*, sausages and minced meat products*, cold cuts and liver pâté, other meat products*, blood and offal*
Fish	oily fish*, lean and semi-lean fish*, unspecified fish*, fish products*, fish sandwich spread, shellfish*, fish offal*
Eggs	whole eggs, egg whites, egg yolks, egg powder
Milk and cream	milk, yoghurt, cream and sour cream, ice cream, cream-based desserts
Cheese	hard cheese, soft cheese, “brown cheese” (traditional Norwegian cheese made from goat’s milk and/or cow’s milk)
Edible fats	butter, margarine, oil, mayonnaise, dressings, mayonnaise-based sandwich spread, other edible fats
Sugar and sweets	sugar, honey, sweet bread spreads, chocolates, sweets
Beverages	coffee, tea, cordials and soft drinks, drinking water, sparkling water, beer, wine, liquor

*unprepared

### Weight, height, basal metabolic rate and physical activity level

Weight and height were recorded by project staff before starting recording dietary intake with the PFD. Weight was measured in light clothing to the nearest 0.5 kg, and height was measured to the nearest 0.5 cm. Body mass index (BMI) was calculated as weight divided by the square of height (kg/m^2^). Estimates of basal metabolic rate (BMR) were calculated from equations based on age, gender and sex [14]. For the majority of the participants, physical activity level (PAL) was measured using the ActiReg® system [10]. For these participants, energy intake (EI) was divided by energy expenditure (EE) and participants having values of EI/EE within the range 0.76–1.24 were defined as acceptable reporters (ARs) [15]. Underreporters (URs) were defined as those having an EI/EE ratio of less than 0.76 while over-reporters (ORs) were those having an EI/EE ratio of more than 1.24. These are the factors suggested by Black for seven days of diet records [15]. For participants not having valid ActiReg® data (worn ActiReg® for less than three days, n = 10) BMR factor was calculated as energy intake divided by BMR and the mean PAL value for the group with ActiReg® data was used (1.71 for men and 1.69 for women) in the equations published by Goldberg and Black [8, 16]. This resulted in a lower cut off of EI/BMR of 1.16 for men and 1.15 for women. The upper cut off was 2.52 for men and 2.49 for women. Subjects with BMR factors lower than the lower cut off were categorized as URs, and subjects with BMR factors higher than the upper cut off were categorized as ORs. Subjects with BMR factors between the lower and the upper cutoff were categorized as ARs.

### Statistical analyses

Statistical analyses were performed using SPSS version 24.0 (SPSS Inc, Chicago, USA) with the exception of weighted kappa coefficients which were calculated using Stata version 15.1 (StataCorp LP, Texas, USA). Results are presented as mean and standard deviations (SD) and were considered to be statistically significant at p<0.05. As relatively few men participated in the study, analyses were not stratified by gender. Differences between means estimated from the PFD and the WR were tested using the paired t-test. Pearson correlation coefficients (r) were calculated to evaluate the agreement between intakes of energy, nutrients and food groups estimated from the two types of recording. As practiced by Hankin et al. [17] correlation coefficients lower than 0.30 were regarded as poor, 0.30–0.49 as fair and 0.50 or higher as good. Individuals were classified into quartiles, and method agreement is expressed as the proportion of participants classified into the same, same or adjacent, and opposite quartile by the two methods. Weighted kappa coefficients were calculated as a measure of agreement for the classification into quartiles. As suggested by Lombard et al. weighted kappa coefficients lower than 0.20 were regarded as poor, weighted kappa coefficients in the range 0.20–0.60 were regarded as acceptable and weighted kappa coefficients higher than 0.60 were regarded as good. Agreement between the two methods was visualized using the Bland and Altman technique[18] plotting the difference between the two methods against the mean of the measurements. This type of plot shows the magnitude of disagreement and spots outliers and possible trends. For the comparison of BMI and energy intakes in URs and ARs, linear regression adjusting for gender was used.

## Results

### Relative validation

Characteristics of the participants are shown in Table 2. Sixty-eight percent of the participants were women. The mean age was 35 years and mean BMI was 24.0 kg/m^2^.

**Table 2:** Characteristics (mean and SD) of the participants.

	Men (n = 37)	Women (n = 77)	All (n = 114)
Age (years)	33 (11)	36 (13)	35 (13)
Height (cm)	181 (7)	168 (6)	173 (9)
Weight (kg)	82 (14)	67 (12)	72 (14)
BMI (kg/m^2^)	24.9 (3.7)	23.6 (4.0)	24.0 (3.9)
BMR (MJ/d)	7.8 (0.7)	6.0 (0.6)	6.6 (1.1)

BMI, body mass index; BMR, basal metabolic rate

Intakes of energy and selected nutrients are given in Table 3. For energy and the majority of the macronutrients, no differences were seen between the methods. However, the percentage of energy (E%) from carbohydrates measured with the PFD was significantly lower compared with the WR (mean difference -1.6 E%, p = 0.001). The E% from total fat was borderline significantly higher with the PFD (mean difference 0.9 E%, p = 0.054). For the micronutrients, the most noticeable differences were the higher intake of vitamin A (mean difference 74 RAE/day, p = 0.03) and calcium (mean difference 74 mg/day, p = 0.006) when recording with the PFD compared with the WR.

**Table 3:** Daily mean (SD) intakes of macro- and micronutrients as assessed by the pre-coded food diary (PFD) and weighed record (WR) (n = 114).

	PFD	WR	p^1^	r_p_^2^
Energy (MJ)	9.5 (2.4)	9.6 (2.5)	0.60	0.67
Fat (E%)	33 (6)	32 (6)	0.05	0.63
SFA (E%)	13 (3)	13 (3)	0.04	0.59
MUFA (E%)	11 (2)	11 (2)	0.77	0.53
PUFA (E%)	6 (2)	6 (1)	0.02	0.58
Protein (E%)	16 (3)	16 (2)	0.15	0.50
Carbohydrate (E%)	47 (7)	49 (7)	0.001	0.76
Added sugar (E%)	9 (4)	9 (4)	0.89	0.56
Fiber, g/MJ	2.3 (0.8)	2.4 (1.1)	0.07	0.72
Alcohol (E%)	4 (5)	4 (4)	0.27	0.73
Vitamin A^3^ (RAE)	957 (439)	883 (428)	0.03	0.65
Vitamin D (μg)	5.1 (4.4)	4.8 (4.7)	0.47	0.65
Tocopherol (mg)	9 (3)	9 (3)	0.80	0.47
Thiamine (mg)	1.4 (0.4)	1.4 (0.4)	0.02	0.71
Riboflavin (mg)	1.7 (0.6)	1.7 (0.6)	0.66	0.65
Vitamin C (mg)	122 (56)	119 (60)	0.60	0.63
Calcium (mg)	957 (351)	883 (305)	0.006	0.64
Iron (mg)	11 (3)	12 (4)	0.43	0.63

MJ, megajoule; E%, percentage of energy; RAE, retinol activity equivalents; SFA, saturated fatty acid; MUFA, monounsaturated fatty acid; PUFA, polyunsaturated fatty acid

^1^Paired samples t-test

^2^Pearson’s correlation coefficient, all p-values <0.001

^3^One person was excluded from analysis because of an extreme intake of retinol due to intake of liver during the PFD recording period

Pearson correlation coefficients for energy and nutrients for the two methods ranged from 0.47 for tocopherol to 0.76 for E% from carbohydrates. The mean correlation coefficient was 0.63. All correlations were statistically significant (p<0.001).

Table 4 shows the extent to which the PFD was able to classify individuals into the same quartile of energy and nutrient intake as the WR. The percentage of individuals classified into the same quartile varied between 33% for tocopherol and E% from protein and 54% for E% from carbohydrates. The mean value was 44%. On average 3% were misclassified into opposite quartiles, ranging from 0% for calcium and E% from carbohydrates to 6% for vitamin A. Weighted kappa coefficients ranged from 0.28 for tocopherol to 0.56 for E% from carbohydrates.

**Table 4:** Percentage of participants classified into the same, same or adjacent, or opposite quartile with the pre-coded food diary (PFD) and the weighed record (WR) (n = 114).

	Classified into same quartile, %	Classified into same or adjacent quartile, %	Classified into opposite quartile, %	Weighted kappa
Energy (MJ)	48	89	3	0.48
Fat (E%)	50	86	1	0.48
SFA (E%)	41	79	1	0.35
MUFA (E%)	42	81	4	0.35
PUFA (E%)	44	82	4	0.36
Protein (E%)	33	82	4	0.29
Carbohydrate (E%)	54	91	0	0.56
Added sugar (E%)	39	85	3	0.36
Fiber, g/MJ	52	90	2	0.52
Alcohol (E%)	53	88	2	0.51
Vitamin A, RAE	42	82	6	0.34
Vitamin D, μg	40	81	4	0.33
Tocopherol, mg	33	82	4	0.28
Thiamine, mg	42	88	1	0.43
Riboflavin, mg	47	92	3	0.49
Vitamin C, mg	44	84	2	0.41
Calcium, mg	46	91	0	0.49
Iron, mg	36	83	1	0.35

E%, percentage of energy; SFA, saturated fatty acid; MUFA, monounsaturated fatty acid; PUFA, polyunsaturated fatty acid, RAE, retinol activity equivalents

**Fig 2:**
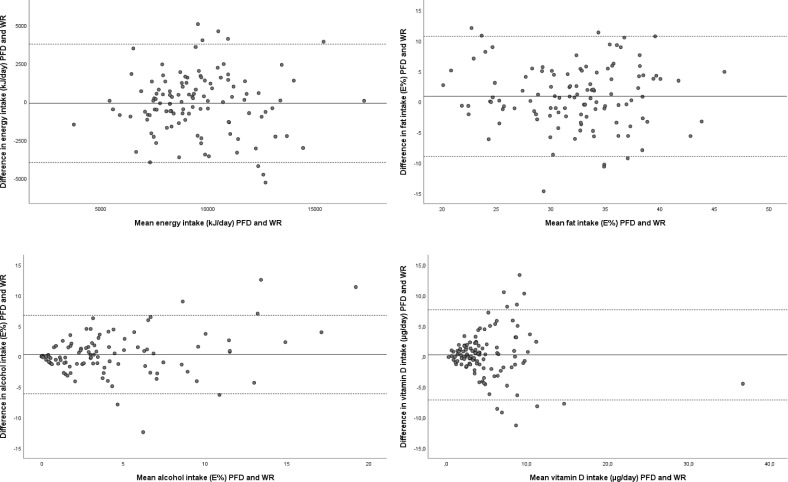
Bland Altman plot showing the difference in energy or nutrient intake measured with the PDF and WR plotted against the mean intake from the two methods. The solid line indicates the mean difference between the methods while the dashed lines indicate ±1.96 SDs. kJ, kilo joule; E%, percentage of energy intake; PFD, precoded food diary; WR, weighed record.

For most nutrients, the Bland Altman plots were similar to the ones shown for energy and fat (Fig 2). Generally, the 95% confidence intervals were wide indicating rather large differences between the PFD and the WR at the individual level. Largely, the differences did not tend to increase with increasing intake. However, the plots for vitamin D and alcohol (Fig 2) showed a tendency towards larger differences between intakes with increasing mean intake. Also for these nutrients wide confidence intervals were seen.

Intakes of the main food groups are shown in Table 5. Significantly different intakes estimated from the two methods were observed for bread for which a lower intake (mean difference -19 g/day, p = 0.001) was observed using the PFD. The intakes of edible fats, cheese and beverages were higher with the PFD than with the WR. Intakes were significantly correlated (p<0.01) for all main food groups for the two methods with correlation coefficients ranging from 0.25 (potatoes) to 0.77 (milk and cream). The mean correlation coefficient was 0.52. No significant differences between total intakes of vegetables, fruits and berries (including maximum 100 g juice/day) were seen with the two methods and intakes were significantly correlated (r = 0.74, p<0.001). The PFD classified 48% of the participants into the same quartiles of total intake of fruits, berries and vegetables as did the WR (weighted kappa = 0.52). None of the participants were misclassified into opposite quartiles according to fruit, berry and vegetable intake.

**Table 5:** Daily mean (SD) intakes of the main food groups as assessed by the pre-coded food diary (PFD) and weighed record (WR) (n = 114).

	All participants (n = 114)			
Food groups (g/day)	PFD	WR	p^1^	r_p_^2^
Bread	149 (82)	168 (78)	0.001	0.73
Cereals	83 (61)	86 (74)	0.63	0.38
Cakes	42 (32)	44 (39)	0.66	0.27
Potatoes	47 (41)	44 (36)	0.52	0.25
Vegetables	150 (73)	162 (102)	0.12	0.63
Fruits and berries	271 (188)	273 (189)	0.86	0.68
Meat	112 (66)	115 (65)	0.67	0.47
Fish	56 (48)	53 (49)	0.44	0.54
Eggs	20 (22)	20 (21)	0.99	0.48
Milk and cream	268 (200)	267 (232)	0.91	0.77
Cheese	47 (30)	41 (26)	0.03	0.50
Edible fats	25 (20)	21 (16)	0.003	0.56
Sugar and sweets	33 (28)	30 (25)	0.29	0.42
Beverages	1802 (829)	1599 (827)	0.001	0.73

^1^Paired samples t-test

^2^Pearson’s correlation coefficient, p<0.001 for all food groups except cakes and potatoes for which p<0.01

### Acceptable reporters (ARs) and underreporters (URs)

**Fig 3:**
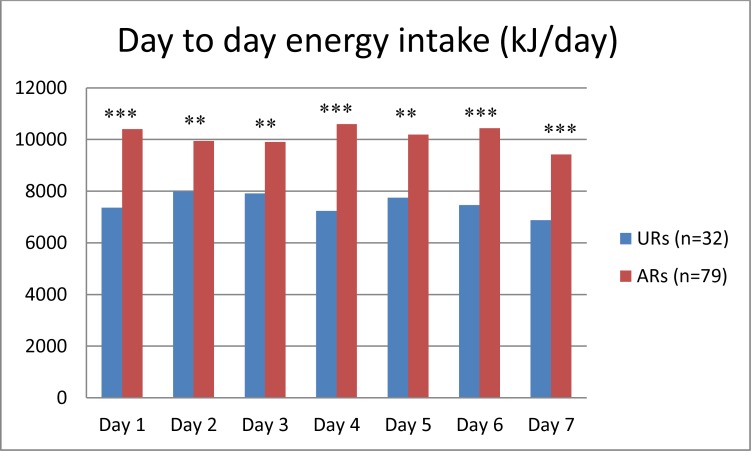
Mean day-to-day energy intake from the pre-coded food diary (PFD) in acceptable reporters (ARs) and underreporters (URs). Three participants classified as overreporters are not included. ***p<0.001 **p<0.01 (linear regression adjusted for gender).

Thirty-two (28%) participants were classified as URs with the PFD while 25 (22%) were classified as URs with the WR. Three participants were classified as overreporters (OR) with the PFD, while one participant was classified as an OR with the WR. Seventeen of the participants classified as URs with the PFD were also classified as URs when recording with the WR. The one person classified as an OR with the WR was also classified as an OR with the PFD. After adjusting for gender, BMI in the group of URs (25.7 kg/m^2^) with the PFD was significantly higher (p = 0.007) than BMI in the group classified as ARs (23.5 kg/m^2^). Fig 3 shows energy intake on each recording day for ARs and URs separately. Energy intake was significantly lower for URs than for ARs for all days of the recording period.

**Fig 4:**
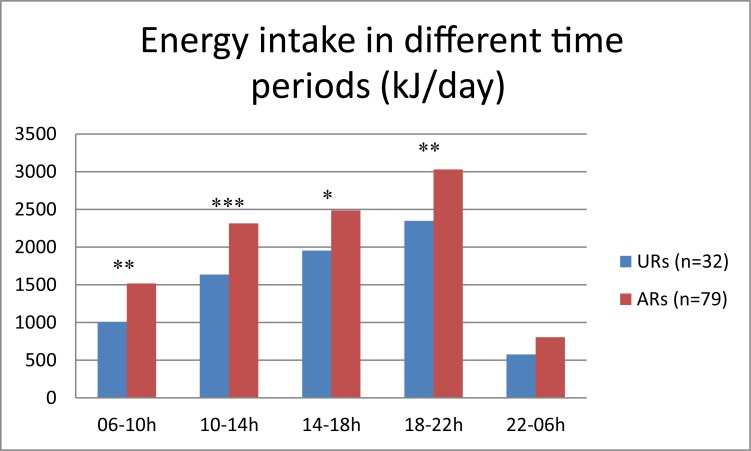
Mean energy intake at different time periods in the pre-coded food diary (PFD) in acceptable reporters (ARs) and underreporters (URs). Three participants classified as overreporters are not included. ***p<0.001 **p<0.01*p <0.05 (linear regression adjusted for gender).

Fig 4 shows energy intake during the five different time periods in the PFD for URs and ARs. Energy intake was significantly lower for URs than for ARs in all time spans except the time span 22:00–06:00h.

## Discussion

In the present study a PFD was validated against WR in a group of Norwegian adults. At the group level, few significant differences were seen in nutrient and food group intake between the methods. At the individual level, correlation coefficients between nutrient intakes estimated from the two methods were generally good [17] with a somewhat larger span in the correlation coefficients for food group intakes. Classification into quartiles was acceptable [19] and few participants were classified into opposite quartiles. Bland Altman plots also showed small differences in intakes estimated from the two methods at the group level, but wider discrepancies at the individual level. When comparing energy intake in ARs and URs, no clear patterns of underreporting at certain recording days or time spans during the day were observed.

### Relative validation

The PFD has previously been validated using WR among Norwegian 9 year olds [20]. Generally, the agreement between the methods was better both at the group level and at the individual level for adults than for children. Fewer significant differences in mean intakes were seen for adults. At the individual level, the median correlation coefficients for energy and nutrients were 0.49 for boys and 0.43 for girls among the 9 year olds, while the mean correlation coefficient in the present study was 0.63. Among the 9 year olds, on average 4% of boys and 7% of girls were classified into opposite quartiles according to intake of energy and nutrients while the corresponding percentage was 3% in the present study. A Danish pre-coded food diary has also been validated against a WR in an adult population [21]. The Danish study found that intakes of nutrients were largely the same by the two methods, while some significant differences in intakes of the main food groups were seen. Further in the Danish study, the correlation coefficients for nutrients varied from 0.16 for vitamin D to 0.72 for dietary fiber, while for the food groups correlation coefficients varied between 0.18 for eggs and 0.88 for coffee. These correlations show a somewhat wider range than in the present study, but similar mean values. The larger variation in correlation coefficients in the Danish study than in the present study may in part be explained by the Danish study including four days of recording rather than seven. In a Swedish study validating a pre-coded food record against WR among adults [5], no differences were seen in energy intake between the methods, and correlation coefficients for intakes of most nutrients were between 0.5 and 0.7. Hence, the mean correlation coefficient of 0.63 observed in the present study is comparable to the results from the Swedish study. Correlation coefficients for intakes of food groups were between 0.35 and 0.74 in the Swedish study. A somewhat greater range of correlation coefficients was observed in the present study but with a similar mean value (0.55 in the Swedish study and 0.52 in the present study). An optically readable version of the same Swedish pre-coded food record has also been validated in a group of elderly Swedish men [22]. In this population energy intake was significantly lower with the pre-coded diary than with the WR, but the proportion of energy intake from each macronutrient was generally similar for the two methods. In a British study [23] comparing several dietary assessment methods to WR, a structured seven days check list with individual portion sizes generally showed higher intakes of the measured nutrients compared to the WR because of larger portion sizes when using the check list. Correlation coefficients for nutrients varied between 0.29 (retinol) and 0.87 (alcohol) with a median correlation coefficient of 0.59, similar to the present study.

When using classification into quartiles, it is expected by chance to get 25% in the same quartile, 62.5% in the same or adjacent quartile and 12.5% in opposite quartiles. The PFD showed an acceptable ability to classify individuals into the same quartile as the WR and few participants were classified into opposite quartiles [19]. According to the guidelines suggested by Lombard et al. [19], the strength of agreement based on the Kappa coefficients was acceptable (acceptable range defined as 0.20–0.60) for all of the studied nutrients. The lowest percentages of classification into the same quartile (33%) were seen for percentage of energy from protein and intake of tocopherol. However, for both these nutrients, 82% were grouped into the same or adjacent quartile, and only 4% were grossly misclassified. The highest percentage of classification into the same quartile for the two methods was seen for carbohydrates (54%), and none of the participants were classified into opposite quartiles for this nutrient. This is interesting as carbohydrate intake (in E%) was significantly lower for the PDF than for the WR. Bread is an important source of carbohydrates and bread intake was significantly lower with the PFD than with the WR. We therefore wanted to see if the difference in carbohydrates was caused by the difference in intake of bread with the two methods. However, carbohydrate intake (E%) was still significantly lower (p = 0.03) when recording with the PFD than with the WR after excluding the contribution from bread, suggesting that bread was not the only cause of the difference. Significant differences in recorded amounts between the two methods were also seen for cheese with a higher cheese intake when recording with the WR. This difference seemed to cause the difference in calcium intake between the methods, as calcium intake from other sources than cheese did not differ between methods (mean difference 17 g, p = 0.41). A more detailed analysis into the subgroups of cheese revealed that the difference in total cheese intake was caused by a higher intake of white cheese (mean difference 6 g/day, p = 0.03) when recording with the PFD. Intake of the traditional Norwegian “brown cheese” was not significantly different between the methods. This may suggest that the portion size for white cheese (20 g per portion) in the PFD was somewhat too large for the present population, while the portion size for “brown cheese” (15 g per portion) was more appropriate. A significant difference in intake of the food group “beverages” was also seen with higher intake estimated from the PFD. When looking into the beverage subgroups, a significant difference was found only for water (mean difference 185 g, p = 0.001) and not for sugar-sweetened beverages, artificially sweetened beverages, coffee and tea or for the alcoholic beverages. The same picture in the photographic booklet was used for estimating the portion size of water as for the other non-alcoholic cold beverages, so it is not clear what caused this difference.

Fruits and vegetables have been linked to several positive health effects [24, 25] and are often a focus for dietary research. Hence, it is important that methods of recording dietary intake show an acceptable ability to estimate fruit and vegetable intake, both at the group level and at the individual level. No significant differences between total intakes of fruits and vegetables recorded with the two methods were observed. About 50% of the respondents were classified into the same quartile of fruit and vegetable intake with the two methods, and no participants were classified into opposite quartiles with a weighted kappa coefficient of 0.52 indicating acceptable agreement between the methods [19].

Some of the differences in nutrient intake observed between the PFD and the WR may be due to real differences in dietary intake during the two periods of recording. Seven days of recording dietary intake has been found unable to correctly rank individuals according to intake of various nutrients, for instance retinol, carotene and polyunsaturated fatty acids [26–28]. Thus, it is not unexpected that somewhat different results between methods were observed for some of the nutrients.

### URs vs ARs

It is well recognized that misreporting of energy intake is a serious problem in dietary surveys [7–9]. This was also the case for the present study as 28% of the participants were found to be URs with the PFD. BMI among URs was significantly higher than among ARs. It has been shown on numerous occasions that underreporting tends to be more pronounced in overweight individuals [29, 30]. Underreporting may consist of undereating in the period of recording, omitting consumed food items from the record or a combination of the two. In this study, the PAL value used for estimating the degree of underreporting was obtained from registration with the ActiReg®. Due to the social desirability of being physically active, it is possible that part of the observed discrepancy between energy intake and energy expenditure was due to the participants being unusually active during the registration period without adjusting their energy intake to the increased activity level. This tendency has been found in another Norwegian study validating energy intake from the food diary against ActiReg® in a group of 13 year olds [13].

The comparison of energy intake between ARs and URs from day to day and at different time points on each day did not show any clear patterns of underreporting at certain recording days or time spans during the day. Hence, underreporting did not seem to be caused by a “burn out” effect where the participants get tired of the recording and report fewer foods on the last days of the period or at the end of each recording day. This is in contrast to the findings by Lillegaard et al. [20] in the relative validation of the same PFD in children. In 9 year olds, energy intake was significantly lower for URs compared to ARs on the last two days of the 4-day recording period. Moreover, energy intake was significantly lower for URs compared to for ARs after 10 a.m., but not in the morning hours [20].

Previous studies suggest that some individuals are more likely to underreport dietary intake than others, regardless of the dietary assessment method used [31]. This tendency was also seen in the present study as 68% of the participants classified as URs with the WR also were classified as URs with the PFD.

The PFD has previously been validated in a group of elderly men (aged 60–80 years) against energy expenditure using a physical activity monitor (SenseWear Pro3 armband) [30]. In this study, the average energy intake was 17–18% lower than the measured energy expenditure, and 47–49% of participants were classified as URs. These numbers are considerably higher than for the present study, but the definition of URs, ARs and ORs was somewhat different, possibly explaining some of the differences in percentage of URs.

### Strengths and limitations

Strengths of the present study include the fairly large sample size. Is has been recommended that validation studies in dietary research preferably should include 100–200 participants [32]. In the present study 114 participants completed both the recording in the PFD and the WR. All participants recorded their diet for 7 consecutive days providing dietary information about all the days of the week in each recording period. The long period of data collection (2 years) also included all seasons of the year.

There are some limitations to the study. The participants were volunteers recruited from academic environments and from fitness centers. Hence, our sample was probably more motivated than what could be expected from the population in general.

Twenty-two percent of the participants underreported energy intake with the WR, raising questions concerning the validity of the reference method. However, the results were not substantially altered when excluding the participants classified as URs with the reference method.

The WR has traditionally been regarded as one of the more reliable methods of measuring dietary intake and is often used in validations of less detailed dietary assessment methods [32, 33]. Therefore this method was chosen as the reference method in this study. In validation studies, the errors associated with the test method and with the reference method should be as independent as possible to avoid spuriously high estimates of validity [32]. The PFD and the WR share several of the same features, both methods capture intake prospectively requiring participants to record dietary intake at the time of consumption. This may affect the participants’ intake, both due to social desirability and due to convenience. Still, the purpose of the present comparison was to study how the PFD compared to another established method, and the results show that although the PFD involves substantially less work for both participants and researchers than the WR, the obtained results were quite similar. The suitability of the PFD will however depend on the research question that is addressed. It is for instance not suitable for answering questions about details on which kinds of meat that are used or specific ingredients in predefined dishes and casseroles as these foods are not specified in great detail in the PFD. Each researcher will have to go through the food selection in the PFD to check if the level of detail is sufficient for the purpose of the study. Likewise, the PFD might be more suited for some subgroups of the population than others. The food selection covers mostly Norwegian foods, and persons eating foods that are not included in the PFD are likely to find it more difficult to use. It is however possible to record food items not listed in the PFD in open ended alternatives.

## Conclusion

The overall results of this relative validation study showed very similar energy intakes and few differences in nutrient intake between the PDF and the WR at the group level. However, the percentage of energy from carbohydrates was lower with the PFD then with the WR, while the intakes of vitamin A and calcium were higher. With regard to the food group intakes, the PFD showed a higher intake of cheese, edible fats and beverages and a lower intake of bread compared to the WR. At the individual level, classification into quartiles was acceptable for all nutrients, with lowest agreement for percentage of energy from protein and tocopherol. The correlation between nutrient intakes was generally good, with somewhat more variation for correlation between intakes of food groups. Because of the reduced work load on both participants and researchers compared to the WR the PFD seems to be a suitable alternative to the WR. The suitability of the PFD will have to be considered for each specific group of persons it is to be used for, including food selection and portion sizes.
